# Polysaccharide-Based Multilayer Nano-Emulsions Loaded with Oregano Oil: Production, Characterization, and In Vitro Digestion Assessment

**DOI:** 10.3390/nano11040878

**Published:** 2021-03-30

**Authors:** Luz Espinosa-Sandoval, Claudia Ochoa-Martínez, Alfredo Ayala-Aponte, Lorenzo Pastrana, Catarina Gonçalves, Miguel A. Cerqueira

**Affiliations:** 1 School of Food Engineering, Universidad del Valle, 76001 Cali, Colombia; luz.a.espinosa@correounivalle.edu.co (L.E.-S.); claudia.ochoa@correounivalle.edu.co (C.O.-M.); alfredo.ayala@correounivalle.edu.co (A.A.-A.); 2 International Iberian Nanotechnology Laboratory, Av. Mestre José Veiga, 4715-330 Braga, Portugal; lorenzo.pastrana@inl.int (L.P.); miguel.cerqueira@inl.int (M.A.C.)

**Keywords:** encapsulation, nanotechnology, high energy method, modified starch, polyelectrolytes, layer-by-layer

## Abstract

The food industry has increased its interest in using “consumer-friendly” and natural ingredients to produce food products. In the case of emulsifiers, one of the possibilities is to use biopolymers with emulsification capacity, such as octenyl succinic anhydride modified starch, which can be used in combination with other polysaccharides, such as chitosan and carboxymethylcellulose, in order to improve the capacity to protect bioactive compounds. In this work, multilayer nano-emulsion systems loaded with oregano essential oil were produced by high energy methods and characterized. The process optimization was carried out based on the evaluation of particle size, polydispersity index, and zeta potential. Optimal conditions were achieved for one-layer nano-emulsions resulting in particle size and zeta potential of 180 nm and −42 mV, two layers (after chitosan addition) at 226 nm and 35 mV, and three layers (after carboxymethylcellulose addition) of 265 nm and −1 mV, respectively. The encapsulation efficiency of oregano essential oil within nano-emulsions was 97.1%. Stability was evaluated up to 21 days at 4 and 20 °C. The three layers nano-emulsion demonstrated to be an efficient delivery system of oregano essential oil, making 40% of the initial oregano essential oil available versus 13% obtained for oregano essential oil in oil, after exposure to simulated digestive conditions.

## 1. Introduction

Nano-emulsions are a dispersion of small droplets of a liquid in another immiscible liquid [1]. The two immiscible liquids most widely used in commercial applications are oil and water. Therefore, nano-emulsions are typically oil-in-water (O/W) or water-in-oil (W/O) [2]. Specifically, O/W nano-emulsions consist of small droplets of oil dispersed in an aqueous medium. They are used to increase the solubility and bioavailability of lipophilic bioactive compounds, such as polyphenols, and act as a vehicle to ensure the delivery of these active compounds to a desired site in the body [3]. The droplets in O/W nano-emulsions are typically covered by an amphiphilic hydrophilic emulsifier. The nature of the emulsifier plays a critical role in determining the performance of the nano-emulsion. Therefore, it should be carefully selected for each specific application [4].

The emulsifiers generally used for the development of food-grade nano-emulsions include small molecules, such as Tweens and Spans. Despite their low cost and good efficiency, the food industry has increased its interest in replacing synthetic emulsifiers with natural alternatives to create products with “consumer-friendly” labels [5]. In this sense, there is a trend to use biopolymers, such as proteins or polysaccharides for the preparation and stabilization of nano-emulsions. Although the concentrations required for proteins are low, they tend to become denatured and precipitate due to high processing temperatures and pH fluctuations [6]. Therefore, the use of polysaccharides is preferable. However, only a few have this ability. Octenyl succinic anhydride modified starch (OSA-MS) is a potential candidate, which, in addition to its surfactant capacity, is stable against high temperatures, wide ranges of pH, and ionic forces [7] and can be combined with other polysaccharides, such as chitosan and carboxymethylcellulose (CMC) [8]. Chitosan is a non-toxic, biodegradable, and cationic polysaccharide consisting of D-glucosamine and N-acetyl-D-glucosamine copolymer units linked by glycosidic β-(1–4) bonds [8] with a positive charge due to the presence of amino groups in the backbone. CMC is an anionic, food grade, and biodegradable polyelectrolyte. Its negative charge is attributed to anionic carboxylate groups in the CMC chains. Some works have shown the possibility of using these two polysaccharides in combination to build nano-systems [9].

O/W nano-emulsions are usually used for the encapsulation and delivery of active compounds dispersed in the oil phase, such as essential oils. One of the essential oils that have been used in food applications is oregano essential oil (OEO). OEO exhibits antioxidant [10], antimicrobial [11], anti-carcinogenic, and anti-inflammatory [12] properties that make it a good choice as a preservative and functional compound. Carvacrol and thymol are the two major phenols that constitute the OEO and are mainly responsible for its antioxidant activity [10]. However, the composition of the OEO may vary as a result of oxidation, chemical interactions, or volatilization. To avoid this problem, they can be encapsulated before being used in food or beverages [13].

The methodologies and materials used for the encapsulation of essential oils are major factors that influence the final characteristics of the nano-emulsion [14]. Among high-energy emulsification methods, high-pressure homogenization [15], micro-fluidization [16], and ultrasounds [17] are the most used. The use of ultrasounds as an emulsification method has received great attention in recent years thanks to its image as a “green technology” and to its high efficiency, economic performance, and equipment requirements [18,19,20]. Some recent reviews show this is a trend in the area [21,22]. In addition to the primary emulsion, ultrasounds have also been used during the addition of another layer. Some examples of the production of oil-in-water emulsions using ultrasounds, showed that using whey protein and pectin results in emulsions with sizes between 1.69–0.93 μm [23] and whey protein and alginate, depending on the condition used, can reach 836 nm [24]. Some authors, such as Kim et al. (2014) and Abbas et al. (2013), reviewed the use of OSA-MS as an emulsifier for the development of ultrasound-assisted nano-emulsions [25,26], and Abbas et al. (2015) evaluated the methodology of layer-by-layer deposition for the development of a multilayer polymeric nano-system for the nanoencapsulation of curcumin using OSA-MS, chitosan, and CMC [27]. However, the encapsulation ability and performance under digestive conditions were not evaluated. In this work, for the formation of the layers, the saturation method was used where the polyelectrolyte concentration needed to coat the droplets completely can be determined by zeta-potential measurements. In this method, it is important to guarantee that the concentration used is enough to cover all particles, but not too much where the free polyelectrolyte in the continuous phase generates an attractive osmotic force strong enough to overcome the various repulsive forces and cause depletion flocculation [28]. 

Therefore, this work aimed to develop and characterize multi-layer nano-emulsion systems loaded with oregano essential oil (OEO) and evaluate their performance under in vitro gastrointestinal conditions. The nano-systems were characterized for their particle size (PS), polydispersity index (PDI), zeta potential (ZP), and morphology. Fourier transform Infra-red spectroscopy and X-Ray diffraction were used for the chemical characterization.

## 2. Materials and Methods

### 2.1. Materials

The materials include oregano essential oil (100% purity, Gran Velada S.I., Zaragoza, Spain); refined sunflower oil (100% sunflower, Sovena, Portugal) as long chain triglycerides (composition: saturated lipids: 10.96%, monounsaturated lipids: 30.44% and polyunsaturated lipids: 58.60%); sodium octenyl succinic anhydride modified starch (OSA-MS) batch No. 02212563 (gift from Brenntag, Porto, Portugal); partially deacetylated chitosan of medium and low molecular weight, poly (D-glucosamine), average molecular weight of 100 kDa, (degree of deacetylation: 75–85%, Sigma-Aldrich Co, Steinheim, Germany); sodium carboxymethylcellulose (Na-CMC) (EMD Milipore Corporation, Billerica, MA, USA); [sodium anhydride acetate (purity > 99%), hexane (purity > 99%), pancreatine (P7545), swine pepsin (P7012), polycreatine α-amylase (A3176), swine intestinal lipase (L3126), bile salts (P8631), (NH_4_)_2_CO_3_ (207861)] Sigma-Aldrich, St. Louis, MS, USA); glacial acetic acid (purity >99.5%, Fisher Chemical, Pittsburgh, PA USA); [KH_2_PO_4_ (0240), HCl (6081)] J.T. Baker, Portugal; NaHCO_3_ (Merck 6329, Kenilworth, NJ, USA.); [KCl (4936), NaCl (6404), MgCl_2_(H_2_O)_6_ (5833), CaCl_2_(H_2_O)_2_ (2382), NaOH (9141)] Merck, Lisboa Portugal); distilled water, and ultrapure water type 1, generated by a Mili-Q system.

### 2.2. Multilayer Nanoemulsions Production

#### 2.2.1. Solutions Preparation

The OSA-MS solution was mixed at a known concentration in distilled water up to a complete dissolution at 250 rpm and 45 °C. Chitosan and sodium carboxymethylcellulose (Na-CMC) solutions were prepared in an acetate buffer solution at different concentrations up to a complete dissolution at 250 rpm and 20 °C. The acetate buffer solution was prepared in a 500 mL volumetric flask dissolving 3.15 g of sodium acetate anhydrous in distilled water. Then 4.5 mL of acetic glacial acid was added and the volume was adjusted. The pH was carried up to 4.5 with a solution of NaOH 1 M in order to increase the solubility of the polyelectrolytes (chitosan, Na-CMC) and to ensure the positive or negative charge of the electrolytic surface in the solution, respectively.

#### 2.2.2. Nano-Emulsions Production

The nano-emulsion I (NE I) was produced by mixing OSA-MS (5.0, 7.5, or 10 mg/mL) and sunflower oil in a ratio of 95:5 (*v*/*v*), respectively. The solution was homogenized using an Ultraturrax (T 25 digital Ultraturrax^®^ disperser, 120 V, Terra Universal Brand, Portugal) at 14,000 rpm for 2 min (25 °C). Afterward, a coarse emulsion was obtained by applying ultrasonic homogenization at a frequency of 20 kHz (1200 W, JY98 IIIDN, Ningbo Scientz Biotechnology Co., Zhejiang, China) with a 20 mm horn diameter. During the ultrasonic homogenization, a cold-water bath was used to avoid overheating and the temperature of the solution was monitored with a digital thermometer. A power 480 W was used for 7 min with pulsed intervals of 5 s ON and 7 s OFF at 30–35 °C. The optimum concentration of OSA-MS was obtained by means of the optimal concentration established by Abbas et al. [27]. The nano-emulsion II (NE II) was carried out by adding dropwise (2.5 mL/min) 20 mL of chitosan solution (pH 4.5) to 40 mL of NE I. The solution was homogenized using the Ultraturrax at 8000 rpm for 5 min (25 °C). Afterward, ultrasonic homogenization was applied for 7 min with pulsed intervals of 5 s ON and 7 s OFF at 30–35 °C. The optimization of NE II was carried out by testing different concentrations (0.12–7.0 mg/mL) of medium and low molecular weight chitosan. The ranges were taken from Abbas et al. in order to establish the optimal concentration [27]. Nano-emulsion III (NE III) was produced by adding dropwise (2.5 mL/min) 10 mL of Na-CMC solution to 40 mL of NE II. The NE III was homogenized using an Ultraturrax at 8000 rpm for 5 min followed by ultrasonic homogenization at 40% of the maximum power of the equipment during 50 s in a pulsed way (5 s ON and 7 s OFF). The optimization of the NE III was carried out by testing increasing concentrations (0.2–2.0 mg/mL) of Na-CMC. The concentration range was defined based on the work of Abbas et al. [27]. The different process parameters tested for the optimization of each nano-emulsion are presented in Table S1 of the supplementary section.

#### 2.2.3. Nano-Emulsions Loaded with OEO

OEO was dissolved in sunflower oil at different ratios. Later concentrations are presented as 99:1, 95:5, 90:10, 85:15, 75:25, 50:50, and 25:75. The first layer was obtained by mixing 7.5 mg/mL of OSA-MS solution (see Section 2.2.1) and OEO mixed with sunflower oil (long chain triglycerides-LCT) as expressed previously in different ratios and concentrations. Afterward, the procedure described in Section 2.2.2 to obtain NE I, NE II, and NE III was followed. 

Some samples were frozen (−80 °C) and then freeze-dried (−50 °C) under 1.09 Pa for 48 h and stored in the dark at −20 °C until further characterization.

#### 2.2.4. Stability Analysis

NE I, II, and III loaded with 1% of OEO were stored in amber flasks and their stability was assessed at different temperatures (4 °C and 20 °C) for 21 days. Visual observation was performed. Particle size, zeta potential, and polydispersity index were determined using the conditions described in Section 2.3.

### 2.3. Particle Size, Polydispersity Index, and Zeta Potential 

The particle size (PS), calculated as the mean diameter of the nano-system, the polydispersity index (PDI), which is a dimensionless parameter, that indicates the heterogeneity of a sample based on size, and the zeta potential (ZP) or surface charge of NE I, II, and III were measured through dynamic light scattering (DLS) technology using a Horiba Nano SZ (UK) equipment with an He-Ne laser (633 nm). Samples were diluted 200 times in distilled water and agitated before the analysis. The zeta potential (ZP) of the nano-emulsions was determined by measuring the electrophoretic mobility. The PS was expressed in nm and the ZP in mV. Five measurements were made for each sample at 25 °C at a detection angle of 80°.

### 2.4. Encapsulation Efficiency

The encapsulation efficiency (EE) was determined for NE I with a ratio of sunflower:OEO 95:1 after the removal of non-encapsulated OEO. The separation was made using an ultrafilter Amicon-0.5 (Amicon^®^ Ultra—0.5 ML 3 K device, Millipore Corp., Cork, Ireland). Briefly, 0.5 mL of the sample was added to the Amicon^®^ and centrifuged at 14,000 × *g* for 15 min. After centrifugation, free oregano in the filtrate was assessed through the absorbance measurement at 276 nm (maximum absorption peak) [13] for the EE calculation. A calibration curve was prepared using different concentrations of OEO in hexane (range 2.5 × 10^−5^–1.0 × 10^−4^ mL/mL hexane). The absorbance values obtained at 276 nm were plotted (Abs = 2468.7 concentration OEO (mL/mL hexane) + 0.0575, R^2^ = 0.9929). Absorbance measurements were carried out in a UV-Vis spectrophotometer (Cary 4000 UV-Visible Spectrophotometer, Biocompare, Portugal). The EE was calculated as follows.

EE (%) = [(OEO_total_ − OEO_free_)/OEO_total_] × 100 (1)

where OEO_total_ is the total amount of OEO present at the beginning and OEO free is the OEO that has not been encapsulated, quantified in the filtrate.

### 2.5. Morphological and Chemical Characterization

#### 2.5.1. Transmission Electron Microscopy

Nano-emulsions were evaluated morphologically by transmission electron microscopy (TEM). In total, 20 µL of a diluted sample (100×) was deposited onto TEM grids (ultra-thin carbon film on Lacey carbon support film, 400 mesh, Copper, Ted Pella Inc., Redding, CA, USA) and the liquid excess was removed with a filter paper. The samples were observed using a JEM-2100 transmission (JEOL, Tokyo, Japan) electron microscope operating at a 200 kV accelerating voltage. 

#### 2.5.2. Fourier-Transform Infrared Spectroscopy (FTIR) 

FTIR spectra of the dried nano-emulsions were recorded with a Bruker FT-IR VERTEX 80/80v (Boston, MA, USA) in Attenuated Total Reflectance mode (ATR) with a platinum crystal accessory in the wavenumber range of 4000–400 cm^−1^, using 16 scans at a resolution of 4 cm^−1^. Before analysis, an open bean background spectrum was recorded as a blank.

#### 2.5.3. X-ray Diffraction 

X-ray diffraction patterns of the films were analyzed between 2θ = 10° and 2θ = 50° with a step size of 2θ = 0.02°/min with a Cu source, X-ray tube (k = 1.54056 Å) at 45 kV, and 40 mA in an X-ray diffraction instrument (X’Pert3 Powder, PANalytical, Almelo, The Netherlands).

### 2.6. Simulated In Vitro Gastric and Intestinal Conditions 

Nano-emulsions loaded with OEO (NE I, II, and III) and OEO in oil were tested regarding their stability under digestive conditions to understand the role of each layer on the OEO protection. The conditions (pH, electrolytes, and enzymes) that mimic the physiological environment in the stomach and intestine were followed, according to Minekus et al. [29]. Simulated gastric fluid (SGF) and simulated intestinal fluid (SIF) were prepared 1.25 times concentrated, considering the later dilution (4:1) with enzymes and CaCl_2_ added in the day of use to avoid precipitation. Quadruplicate were performed for each condition.

For the gastric phase, 5 mL of the sample were added to 4.0 mL of SGF, 7.5 mL of SGF with lecithin (10 mg/mL), 1.5 mL of pepsin (10 mg/mL in SGF), 5 μL of CaCl_2_, and 1.995 mL of milli-Q water. The pH was adjusted to 3.0 (using HCl 1 mM) and left in constant agitation in a thermomixer (Eddingtons Mincer Pro, 86002, Berkshire) at 500 rpm (37 °C) for 2 h. After that time, 2 mL of sample were collected and frozen.

The intestinal phase was prepared by adding to the previous mixture, 11 mL of SIF, 5.0 mL of pancreatine (70 mg/mL in SIF), 2.5 mL of bile salts (40 mg/mL in SIF), 40 μL of CaCl_2_, 150 μL NaOH, and 1.31 mL milli-Q water. The pH was adjusted to 7.0 (using NaOH 1 mM) and left in constant agitation in a thermomixer (Eddingtons Mincer Pro, 86002, Berkshire) at 500 rpm (37 °C) for 2 h. Then, the samples were centrifuged at 14,000× *g* and 8 mL of the supernantant was collected and frozen. This sample was considered the “micellar phase” in which the OEO was solubilized and in physiological conditions ready for intestinal absorption. In some samples, a top layer of non-digested oil was observed and discarded. 

The concentration of the phenolic compound present in each digestion phase was determined by solvent extraction following the methodology proposed by Wright et al. [30]. Briefly, a sample aliquot of 0.5 mL was taken, and 0.5, 3.0, and 1.0 mL of ethanol, acetone, and deionized water were added, respectively, and vortexed for 10 s after the addition of each solvent. Subsequently, 2 mL of hexane were added. The vessel was inverted 10 times. After 5 min of waiting to allow phase separation, the bottom layer of hexane containing solubilized OEO was collected while the top layer was mixed with another 1 mL of hexane and the same procedure was followed, to ensure efficient extraction of phenolic compounds. Hexane extraction was carried out in triplicate for each sample.

For absorbance quantification, hexane was evaporated using a rotary evaporator at 35 °C for 12 min with vacuum pressure. In total, 1 mL of fresh hexane was added and the sample absorbance was measured at 276 nm. The concentration of the compound was quantified according to the following equation.

% OEO _simulated phase_ = (mass OEO _simulated phase_/mass OEO _initial_) × 100 (2)

### 2.7. Statistical Analysis

The information is presented as the average ± standard deviation. The experimental values were analyzed by an analysis of variance (ANOVA) using the statistical program Minitab XVIII. For all analyses, the difference in significance between averages was determined at a confidence level of 95% (*p* < 0.05). To identify treatments with significant differences, a Tukey mean comparison test was applied. The graphics were made using the program Origin Pro 2018 (OriginLab Corporation, Northampton, MA, USA).

## 3. Results and Discussion

### 3.1. Nanoemulsions without OEO

#### Effect of the Polysaccharide’s Concentration and Chitosan Molecular Weight

The NE I was produced using OSA-MS, in which, due to its amphiphilic character, is able to act as an emulsifier. Three concentrations of OSA-MS were evaluated (5.0, 7.5, and 10.0 mg/mL) for NE I production. It was verified that, for 5 mg/mL of OSA-MS, the NE ZP did not reach values lower than −30 mV and, for concentrations higher than 7.5 mg/mL of OSA-MS, the addition of the second layer (using the concentrations presented in Table S1 of the supplementary section for NE II) the NE did not reach a positive charge and, due to that, the third layer was not able to form, making NEs I and II very unstable. Thus, an OSA-MS concentration of 7.5 mg/mL was selected, resulting in PS of 180.98 ± 0.94 nm, a PDI of 0.25 ± 0.02, and a ZP of −42.02 ± 1.32 mV. These results are in agreement with Abbas et al. [27]. The negative charge (−42.02 ± 1.32 mV) of the NE is explained by the carboxyl group of OSA-MS, which guarantees that the deposition of an additional layer could happen by electrostatic interactions between two charged polymers. However, it was seen that the formation of the layer by dropwise addition resulted in higher PS and PDI (results not shown), which can be explained by the presence of small crystalline particles in OSA-MS attributed to the crystalline structure of OSA-MS (starch granules have a layered organization with alternating amorphous and semi-crystalline regions). Due to the formation of the multilayer needing to be assisted with a high energy method, such as ultrasounds will allow one to obtain a more homogeneous nano-system. 

**Figure 1:**
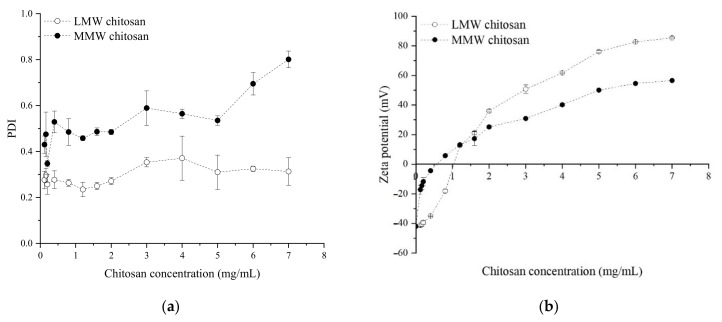
Effect of increasing concentrations of low and medium molecular weight chitosan on the (**a**) polydispersity index (PDI) and (**b**) zeta potential (ZP) of NE II.

In order to evaluate the effect of concentration and molecular weight of chitosan in the production of NE II, thirteen concentrations of both low and medium molecular weight (LMW and MMW, respectively) chitosan were evaluated. PS values of NEs coated with LMW chitosan vary between 200–380 nm and between 190–400 nm for coatings with MMW chitosan (Table S2 supplementary section). Results showed that there was a statistically significant difference (*p* < 0.05) of PS for NEs coated with different molecular weight chitosan at a given concentration and between the NEs at different concentrations. Moreover, there was also a statistically significant effect (*p* < 0.05) of PDI and ZP for NEs coated with different molecular weight chitosans and concentrations (Figure 1). NE II coated with LMW chitosan presented the best values of PDI and ZP being 2.0 mg/mL of concentration that allowed us to obtain an NE with minimum PS and PDI and, at the same time, had an optimal surface charge for the stability of the nano-system (more than 30 mV). It was observed that, when the concentration of chitosan increases, the PDI also increases (Figure 1a). This behaviour agrees with Esmaeilzadeh-Gharedaghi et al. [31] who reported an increase of the PS and PDI with the concentration of chitosan during the production of chitosan nanoparticles, using ultrasounds. This behaviour can be explained by the presence of primary hydroxyl groups in the chitosan that interact with the secondary hydroxyl groups present in the NE I, leaving the amino groups free forming complexes in the solution, increasing the ZP and allowing us to form a random structure that increases the size and PDI values [8].

The use of low molecular weight chitosan on NE II leads to lower PDI values (*p* < 0.05) and more stable ZP than medium molecular weight (Figure 1). An explanation for this can be that low molecular weight chitosan generates more organized molecules in the interphase and, thus, increases the surface charge. Additionally, low molecular weight chitosan has a higher solubility in buffer solution (at the same temperature, pressure, and concentration) [32], so there are no traces of undissolved particles that could increase the PS and PDI. In addition, it is well known that the energy input delivered to the coarse emulsion through sonotrope generates intensive disruptive forces due to acoustic cavitation, so this phenomenon has the potential of reducing larger O/W drops into nano-sized droplets and decreasing the PDI [27], increasing the stability of the well-structured formed nano-emulsions. The second layer formed by chitosan presented a ZP of +44.91 ± 0.71 mV.

After the optimization of the NE II, leading to the use of LMW chitosan at a concentration of 2.0 mg/mL, the NE III was developed and six different concentrations of carboxymethylcellulose (CMC) were evaluated (Table S3 of the supplementary information section). It was observed that the PS and PDI values of the NEs increased while the ZP values decreased for higher concentrations of CMC. The surface charge reached a negative value at a concentration of 2.0 mg/mL of CMC. It was observed that, for higher concentrations of CMC, the values of PDI increase significantly and the system became unstable. This behaviour can be explained by the free polyelectrolyte in the continuous phase that could lead to the depletion flocculation that occurs when the free CMC in the continuous phase generates an attractive osmotic force strong enough to overcome the various repulsive forces and, thus, lead to flocculation [28]. Thus, the optimum CMC concentration taking into account the values of PS (306.18 ± 92.70 nm), PDI (0.76 ± 0.09), and ZP (−6.43 ± 0.58 mV) was considered 2.0 mg/mL with a statistically significant effect (*p* < 0.05). 

### 3.2. Nanoemulsions Loaded with OEO

#### 3.2.1. Effect of OEO on Nano-Emulsions 

Table 1 shows that ZP decreases for higher OEO concentrations. The ZP values changed from −42.02 ± 1.32 mV to −36.15 ± 0.17 mV, from 44.91 ± 0.71 mV to 42.31 ± 0.31 mV, and from −6.43 ± 0.58 mV to −0.92 ± 3.43 mV for NE I, II, and III, respectively. The change of ZP from negative to positive values and again to negative values clearly shows the effect of the polyelectrolyte on the layer formation. The loaded nano-emulsions were evaluated for different concentrations of OEO. It was observed that the incorporation of OEO (1%) decreases the PS of NE I, II, and III (Table 2), being the most significant from 306.18 ± 92.70 to 264.51 ± 81.20 nm for NE III.

In NE I, the incorporation of OEO also resulted in a statistically significant effect (*p* < 0.05) on the PS. This is probably due to the replacement of the long chain triglyceride present in sunflower oil by the triglycerides of shorter chains present in the OEO [33]. In addition, the ZP values of NE I decreased (*p* < 0.05) as the OEO concentration increased. This behaviour can be explained by the adsorption of OSA-MS to the droplet interphase due to its branched polymeric structure [27]. The starch chains were re-associated as double helices in a variable ordered semi-crystalline structure [34]. Thus, it provided higher stability to NE I through steric hindrance, as the OEO concentration increased.

In NE II (Table 2), the PS also decreased (*p* < 0.05) for higher concentrations of OEO. Chitosan was adsorbed on the surface of previously formed NE I, forming the second layer. However, it is likely that not all the chitosan present in the solution was adsorbed, leaving traces of chitosan not adsorbed and leading to an increase in the PDI. Furthermore, for higher OEO concentrations, a decrease (*p* < 0.05) of the ZP (Table 2) is observed, suggesting that, as the OEO increased, the stability of NE II was reduced. 

In NE III (Table 2), the Na-CMC layer does not allow the formation of a stable nano-emulsion and only 1% of OEO allows the formation of three layers of nano-emulsion. The values of PS and PDI obtained were 264.51 ± 81.20 nm and 0.64 ± 0.04 nm, respectively. This fact might be due to the ability of oil molecules to migrate from smaller droplets to larger droplets through the aqueous phase, known as the Ostwald ripening effect [35]. Essential oils containing carvacrol and thymol are specifically prone to Ostwald ripening due to their relative solubility in water because of their high content on OH groups. In fact, Ziani et al. (2011) found that thyme oil-in-water nano-emulsions stabilized by a non-ionic surfactant were highly unstable to droplet growth and phase separation. They attributed this phenomenon to Ostwald ripening [36]. As for the ZP, it remains negative but very close to zero (−0.92 ± 3.43 mV).

#### 3.2.2. Encapsulation Efficiency 

Encapsulation Efficiency (EE) was determined to the optimal OEO concentration (1%) for all the three nano-systems and a mean value of 97.12 ± 0.01% was obtained. Several authors have determined the EE of bioactive compounds within nano-systems. Matos et al. [34] compared the EE of resveratrol-loaded nano-emulsions stabilized with Tween 20 or OSA-MS, and obtained an EE of 63.4 ± 0.7% or 98.3 ± 2.2%, respectively. Carneiro et al. [37] used a combination of maltodextrin and four types of wall materials (OSA-MS, whey protein concentrate, gum arabic, and dextrin) to encapsulate flaxseed oil. They found that the type of wall material has a statistically significant influence on the EE. They obtained an EE ranged between 62.3% and 95.7%. The lowest value was obtained for the combination of maltodextrin and whey protein concentrate. The highest value was obtained for the combination of maltodextrin and OSA-MS. This combination resulted in particles with considerably lower surface oil than those prepared with the other ones. Yoksan et al. [38] encapsulated ascorbyl palmitate (a source of vitamin C in the form of lipid-soluble ester) with chitosan and obtained an EE between 39% and 77%. Therefore, it could be concluded that the EE of the OEO in the system developed in this work is relatively high when compared with other surfactants or encapsulating materials. 

### 3.3. Morphological and Chemical Characterization 

#### 3.3.1. Transmission Electron Microscopy (TEM)

**Figure 2:**
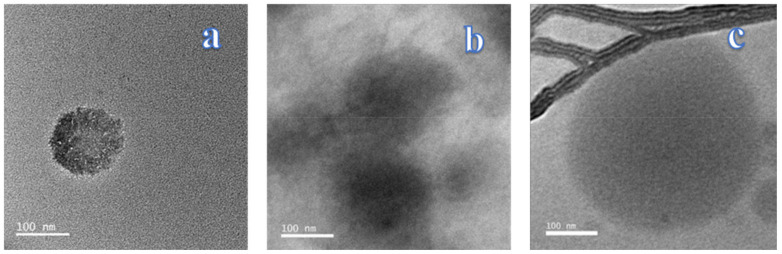
Transmission Electron Microscopy (TEM) images of OEO-loaded NE I (**a**) NE II (**b**) and NE III (**c**).

Figure 2 shows TEM images of the nano-systems obtained. Figure 2a shows the NE I. Here, rounded edges and an irregular or jagged perimeter can be noticed. This reflects an emulsion that is stabilized by OSA-MS semi-crystalline particles that are adsorbed onto the oil interface [34]. In addition, a surrounding white cover, negatively charged according to DLS studies, that gives a start to the polymerization can be observed [27]. After the chitosan addition (Figure 2b), an encapsulation of small OEO-loaded OSA-MS spherulites previously formed in the first layer can be seen. In Figure 2c, all the three layers gathered bordering the capsule like a cordon or thick rope that surrounds a much more extended nucleus that can be observed. This rope-like surrounding has an intense black colour likely due to higher electron density in the interface [39].

In DLS measurements, PS of 180.96 ± 0.64 nm, 252.57 ± 56.79 nm, and 264.51 ± 5.58 nm, for NE I, II, and III, respectively, were obtained. TEM images showed a particle size smaller (<100 nm) than the DLS analysis for NE I. Similar results (>200 nm) were obtained from both techniques for NE II and III. Although not exactly the same, likely due to the dehydration that took place during the preparation of the samples and that only some images were determined, but it can be said that PS obtained by TEM confirms the DLS results. 

#### 3.3.2. FTIR and XRD Analysis

**Figure 3:**
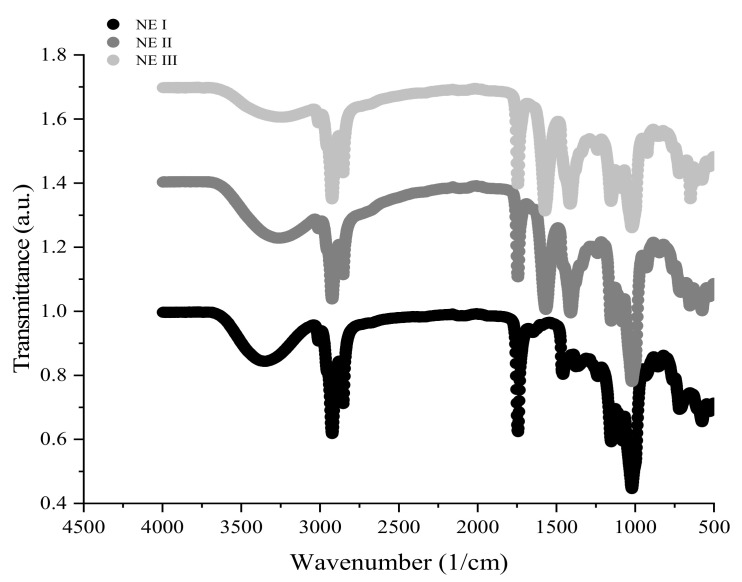
FTIR of 1% OEO-loaded NE I, II, and III.

Figure 3 shows the FTIR spectra of the OEO-loaded (1%) NE I, II, and III. A more pronounced peak at 649 cm^−1^ was found in NE III. This peak is typical of CMC due to the -CH-O-CH2 stretching vibration [40]. A clear characteristic peak at 1020 cm^−1^ indicates the trans-conjugated alkene C-H out-of-plane, which shows that OEO had been successfully incorporated in the nano-emulsions [41]. A peak at 1151 cm^−1^ in NE I and II and at 1153 cm^−1^ in NE III was assigned to symmetric COO− stretching vibration and glycosidic linkages in the polysaccharides [42]. It can be seen that a peak of N-H bending in NE II (chitosan) at 1571 cm^−1^ and at 1569 cm^−1^ in NE III (Na-CMC) indicating that a layer had been added. A strong characteristic peak at around 1743 cm^−1^ in the spectra of all the layers was assigned to the C=O stretching modes [41]. A band correspondent to saturated carbon-hydrogen bonds C-H, typical for organic compounds, was present in the spectra of all NEs. The bands appearing between 2750 and 3000 cm^−1^ are due to stretching vibrations of C–H bond in –CH_2_ (2923 cm^−1^) and –CH_3_ (2856 cm^−1^) groups, respectively [43]. The broad band ranging between 3100 and 3500 cm^−1^ is attributed to O-H stretching vibration. This band was reduced as the layers were added. Thus, NE I (OSA-modified starch molecules) changed from 3367 cm^−1^ to 3292 cm^−1^ when the second layer was added (NE II) and, finally, to 3286 cm^−1^ when the third layer was added (NE III), which is possibly related to the incorporation of each layer. Overall, these FTIR results suggest that OSA-modified starch can encapsulate OEO as well as form a multi-layer nano-system using chitosan and CMC [41].

**Figure 4:**
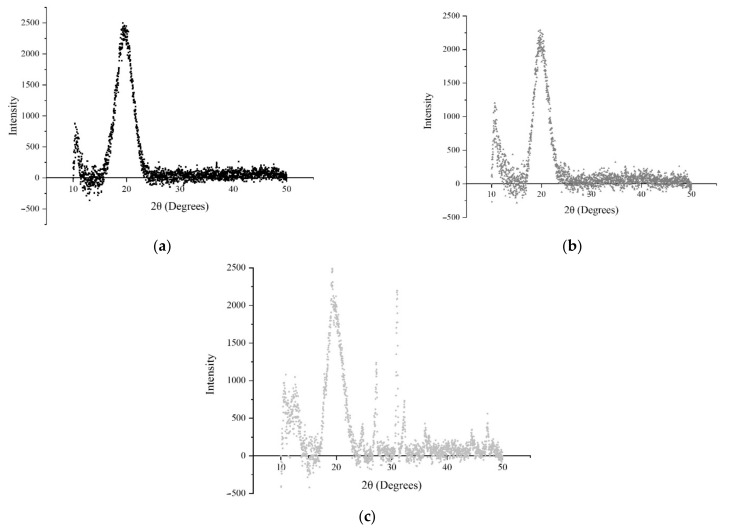
X-ray diffraction of OEO-loaded (1%) nano-emulsions (**a**) NE I, (**b**) NE II, and (**c**) NE III.

X-ray diffraction was used to study the crystalline structure of NE I, II, and III. Figure 4 shows that the three nano-emulsions exhibited diffraction peaks at Bragg angles (2θ) 10° and 20°. In NE I (Figure 4a), the occurrence of these peaks suggested that the crystalline structure of OSA-modified starch follows a typical A-pattern structure [44]. When starch was heated with water, the order-to-disorder transition occurred. During the first portion of the phase transition, water absorbed by starch granules increased the mobility of starch polymers [45]. In NE II (Figure 4b), it is well known that chitosan is a polysaccharide that is partially crystalline due to its regular chain. The reflection around 10° (peak I) indicated the presence of the crystal form I and can be attributed to the hydrated crystalline structure of chitosan. The strongest reflection at 2θ around 20° (peak II) corresponded to the crystal form II and was reported to be the indication of the relatively regular crystal lattice in chitosan [43,46]. In NE III (Figure 4c), the presence of several peaks can be associated with a micellar cubic phase due to the COO from the polyion and, therefore, it is expected that micelles will be more spherical in the presence of CMC. The peaks found at 10° and more pronounced at 20° are obtained by the polymeric complexes previously formed and the existence of these peaks indicates that some order already existed inside these complexes. Thus, the micelles formed during the first and second layers help retain the organisation of the added phase. The micelles’ formation is suggested to be that OSA-MS locates uniformly around the cross-section interface of oil/water, which is likely distributed in the interior amorphous domains of amylopectin molecules, acquiring the ability to stabilize oil-in-water emulsions by combining the hydrophobicity of the octenyl group with the hydrophilic carboxyl or sodium carboxylate groups [47] by achieving a crystalline structure shown as the strong A-type peak at 20° (Figure 4a). After the addition of NE II, OSA groups first attack the surface and then some pores form with little change in the crystalline pattern (Figure 4b) where the LMW chitosan surface attachment depends on the degree of deacetylation and molar mass and is mainly due to jamming at the interface caused by the polymer orientation. The addition of the second layer, for instance, could have decreased the specific viscosity and increased the surface tension and the critical micellar concentration (CMC) of OSA-MS when compared to the single OSA-MS solutions [48]. Thus, the LMW chitosan and Na-CMC interact through the formation of inclusion complexes by generating physical crosslinking points between LMW chitosan tails (RC(O)NH_2_) and Na-CMC helix (RCO_2_Na or CH_2_CO_2_Na) setting a crystal plane for which the secondary radiation displays other intensities as a result of a constructive interference (Figure 4c) [49,50]. When Na-CMC is added to an oppositely charged polymer, it binds due to the strong electrostatic attraction between both species, thus, displacing the counterions into the solution and obtaining other peaks (such as those found at 25° and 30°) [51].

### 3.4. Stability of OEO Loaded-NE

**Figure 5:**
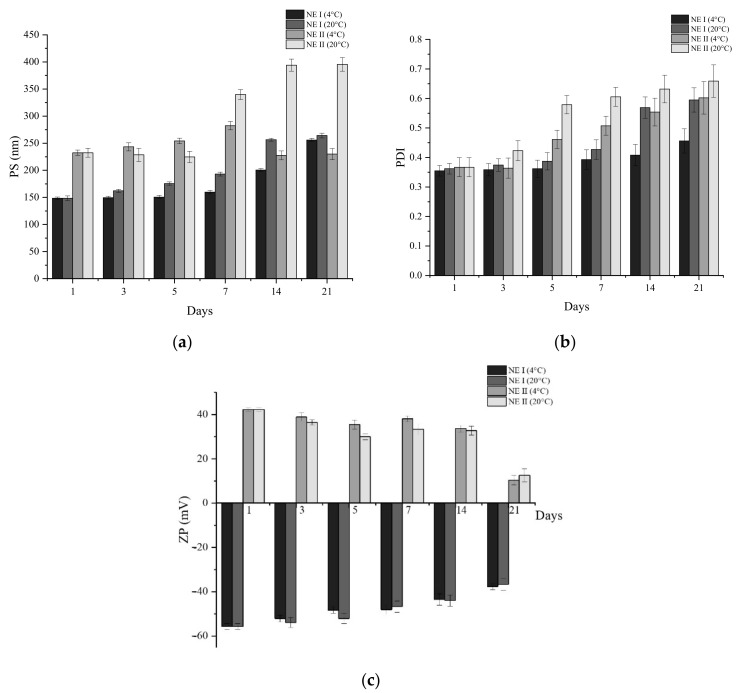
(**a**) Particle size (PS), (**b**) polydispersity (PDI), and (**c**) zeta potential (ZP) of loaded NE I and NE II, as a function of time for different storage temperatures.

Based on the results obtained, it was decided to perform the stability, morphological, and chemical tests of the NEs with 1% of OEO concentration. Figure 5 shows the effect of storage temperature (4 and 20 °C) on PS, PDI, and ZP of loaded NE I and II for 21 days. Table S4 of the supplementary section shows the same effect on the loaded NE III. It was observed that the PS and PDI values increased during the storage time for both temperatures. However, when stored at 4 °C, the values of PS and PDI were more stable during the evaluation period when compared with 20 °C. Thus, the stability of loaded NE I and II was higher (*p* < 0.05) at 4 °C than 20 °C. The increase of the PS and PDI values are followed by the formation of a small cream layer on the top of the NE, known as Ostwald ripening, which was noticed visually. Guerra-Rosas et al. [52] assessed the stability of OEO nano-encapsulated by micro-fluidization, using high methoxyl pectin and a non-ionic surfactant (Tween 80), and observed that PS and PDI increased from 169.30 ± 10.69 nm to 1017.00 ± 198.40 nm and from 0.38 ± 0.04 to 0.41 ± 0.02, respectively, during storage. They also obtained a separation of a serum and attributed this phenomenon to differences between density of a continuous and dispersed phase. Additionally, in the present work, a reduction in the ZP of NE I, II, and III was observed (Figure 5c), and no statistically significant differences (*p* < 0.05) were present between temperatures for all of them. This behaviour confirms the destabilization of loaded NE I, II, and III during storage. However, the ZP remained closer to zero as the OEO concentration increased, resulting in less stability of the nano-system. This could be explained likely due to the high affinity of water to the polyphenols present in the OEO [53]. The energy delivered to the coarse emulsion generates intensive disruptive forces that reduce large O/W drops into nano-sized droplets [27]. The reduction of the droplet’s size increases the stability of the nano-emulsions formed. Klang et al. [54] reported that, when PS and distribution (PDI) remain constant for an extended period of time, the formulation is considered physically stable.

### 3.5. Release of OEO under In Vitro Gastric and Intestinal Conditions

**Figure 6:**
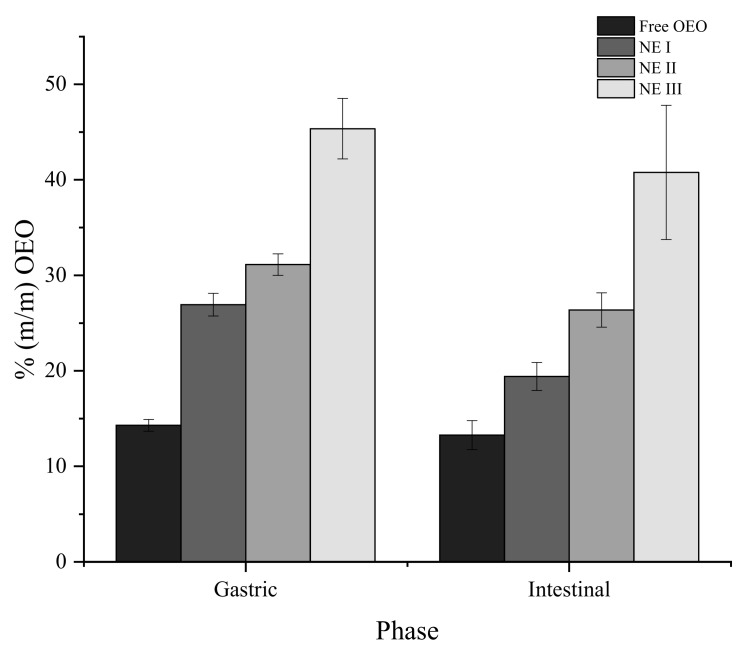
OEO (% m/m) present in the gastric and intestinal phases after the simulated digestion of NE I, II, and III and free OEO. *p*-values of 3.23 × 10^−4^ and 1.77 × 10^−4^ for the gastric and intestinal phases, respectively.

Efficient delivery systems of lipophilic molecules should result in high fractions (%) of bioactive available for intestinal absorption (bioaccessibility). There are several factors determining the absorption of orally ingested lipophilic molecules such as particle size and transport medium, among others [1]. The lipophilic bioactive must be released from the matrix and incorporated into mixed micelles, which are formed primarily of co-ingested lipid digestion products and bile salts in the aqueous digestive phase. Figure 6 shows that the amount (% *m/m*) of OEO present in each digestion phase was NE III > NE II > NE I > free OEO with a statistically significant difference (*p* < 0.05). The results obtained demonstrate that the addition of chitosan and Na-CMC layers affects the amount of essential oil available at the end of each digestion phase (gastric and intestinal). This behaviour can be explained by the protection of the bioactive compound during the pH changes and enzyme action, leading to an increase of the fraction found in each simulated phase when comparing with free OEO (without polymeric layers).

Abbasi et al. [55] developed ultrasound-assisted flaxseed O/W nano-emulsions using a synthethic emulsifier (Tween 80) and whey protein (WP), sodium alginate (SA), and a mixture of polymers (WP/SA), as encapsulating materials. They indicated that nano-emulsions stabilized with two polymers are more resistant to degradation during their passage through the gastrointestinal environment. Moreover, with these polymers, they obtained an amount of flaxseed oil of 10% after the simulation, lower than the bioaccessibility obtained in the present work (41%). Similarly, Zhang et al. [56] showed that, when two polymers are used (WP/SA) for the formation of carvacrol-loaded microcapsules, the bioactive compound was effectively protected in gastrointestinal pH conditions. The proposed multilayer nano-emulsion appears as an efficient delivery system of OEO reaching 40% of the initial OEO added, after intestinal conditions, when three layers are used.

## 4. Conclusions

Optimal conditions were achieved for the production of OEO-loaded nano-emulsions, using OSA-MS as an emulsifier. Nano-emulsions were obtained with an average particle size of 180 ± 0.94 nm and a zeta potential of −43 mV. After the addition of the chitosan layer (second layer), the surface charge changed to a positive value (+35 mV) and the average size increased to 226 nm. The addition of the CMC (third layer) resulted in a particle size of 265 nm and a negative surface charge of −0.9 mV. The encapsulation efficiency of the nano-emulsion loaded with 1% of OEO was 97.11 ± 0.01%. The behaviour of the 3 systems (NE I, NE II, and NE III) under in vitro gastric and intestinal conditions showed that each polymeric layer influences the OEO bioaccessibility, resulting in a higher value when the three-layer system is used. This study shows how multilayer nano-emulsions can be used as delivery systems for lipophilic bioactive compounds.

## Figures and Tables

**Table 1:** Zeta potential (ZP) of NE I, II, and III for increasing oregano essential oil (OEO) concentrations.

OEO Concentration (%)	ZP (mV) ^1^		
	NE I	NE II	NE III
0	−42.02 ± 1.32	44.91 ± 0.71	−6.43 ± 0.58
1	−36.15 ± 0.17	42.31 ± 0.31	−0.92 ± 3.43
5	−39.70 ± 0.36	38.75 ± 0.53	−3.54 ± 2.52
10	−44.14 ± 0.90	38.54 ± 0.27	−7.62 ± 2.53
15	−46.66 ± 0.54	38.43 ± 0.96	−9.16 ± 0.61
20	−45.68 ± 0.67	37.81 ± 0.26	−11.43 ± 0.51
25	−48.74 ± 0.62	36.22 ± 0.27	−13.98 ± 1.61
50	−47.40 ± 0.97	34.88 ± 0.23	−17.65 ± 4.44
75	−48.64 ± 0.55	27.61 ± 0.24	−18.82 ± 0.92
*p*-value	5.35 × 10^−2^	5.96 × 10^−2^	5.55 × 10^−2^

^1^ The values are presented as the average of five samples ± standard deviation.

**Table 2:** Particle size (PS) and polydispersity index (PDI) of NE I, II, and III for increasing oregano essential oil (OEO) concentrations.

OEO Concentration % (*v/v*)	PS (nm) ^1^				PDI ^1^	
	NE I	NE II	NE III	NE I	NE II	NE III
0	180.98 ± 0.94	227.98 ± 11.39	306.18 ± 92.70	0.25 ± 0.03	0.27 ± 0.48	0.76 ± 0.09
1	180.96 ± 0.64	252.57 ± 56.79	264.51 ± 81.20	0.27 ± 0.02	0.27 ± 0.04	0.64 ± 0.04
5	182.72 ± 0.38	248.64 ± 33.69	>1000	0.27 ± 0.06	0.26 ± 0.10	>1
10	182.72 ± 0.61	236.81 ± 55.83	>1000	0.24 ± 0.02	0.24 ± 0.15	>1
15	181.84 ± 0.15	232.35 ± 55.83	>1000	0.19 ± 0.05	0.29 ± 0.05	>100
20	181.60 ± 0.23	229.38 ± 5.66	>1000	0.20 ± 0.03	0.29 ± 0.06	>100
25	181.50 ± 0.20	220.72 ± 1.55	>1000	0.22 ± 0.02	0.29 ± 0.06	>1000
50	160.70 ± 0.35	215.84 ± 41.74	>1000	0.23 ± 0.04	0.24 ± 0.07	>1000
75	139.82 ± 28.83	212.45 ± 67.62	>1000	0.23 ± 0.05	0.33 ± 0.10	>1000
*p*-value	5.49 × 10^−2^	8.55 × 10^−2^	-	1.53 × 10^−3^	1.16 × 10^−3^	-

^1^ The values are presented as the average of five samples ± standard deviation.

## Data Availability

The data presented in this study are available on request from the corresponding author.

## Supplementary Materials

The following are available online at https://www.mdpi.com/article/10.3390/nano11040878/s1, Table S1: Process parameters and their corresponding levels for the preparation of NE I, II, and III, Table S2. Particle size (PS) of NE II for increasing concentration of low and medium molecular weight chitosan (LMW and MMW, respectively). Table S3. Effect of Na-CMC concentration on the particle size (PS), polydispersity index (PDI), and zeta potential (ZP) of NE III. Table S4. Particle size (PS), polydispersity index (PDI), and zeta potential (ZP) of loaded NE III during time at 4 and 20 °C.
