# Supplementary material for: Polysaccharide-Based Multilayer Nano-Emulsions Loaded with Oregano Oil: Production, Characterization, and In Vitro Digestion Assessment
Source: Nanomaterials (Basel). 2021 Mar 30;11(4):878. doi: 10.3390/nano11040878 (PMC8067034; doi:10.3390/nano11040878)
Supplement: Supplementary file 1 [file nanomaterials-11-00878-s001.pdf]

## Supplementary Material

# Polysaccharide-Based Multilayer Nano-Emulsions Loaded with Oregano Oil: Production, Characterization, and In Vitro Digestion Assessment

**Luz Espinosa-Sandoval [1], Claudia Ochoa-Martínez [1], Alfredo Ayala-Aponte [1], Lorenzo Pastrana [2], Catarina Gonçalves [2,*] and Miguel A. Cerqueira [2]**

[1] School of Food Engineering, Universidad del Valle, 76001 Cali, Colombia; luz.a.espinosa@correounivalle.edu.co (L.E.-S.); claudia.ochoa@correounivalle.edu.co (C.I.O.-M.); alfredo.ayala@correounivalle.edu.co (A.A.A.-A.)
[2] International Iberian Nanotechnology Laboratory, Av. Mestre José Veiga, 4715-330 Braga, Portugal; lorenzo.pastrana@inl.int (L.P.); miguel.cerqueira@inl.int (M.A.C.)
* Correspondence: catarina.goncalves@inl.int; Tel.: +3-51-253-140-112

**Table S1.** Process parameters and their corresponding levels for the preparation of NE I, II and III.

| Nanoemulsion | Process Parameters and Formulation | Levels |
|---|---|---|
| NE I | OSA-MS concentration | 7.5 mg/mL |
| | LMW chitosan concentration | 0.12, 0.16, 0.2, 0.4, 0.8, 1.2, 1.6, 2.0, 3.0, 4.0, 5.0, 6.0 and 7.0 mg/mL |
| NE II | MMW chitosan concentration | 0.12, 0.16, 0.2, 0.4, 0.8, 1.2, 1.6, 2.0, 3.0, 4.0, 5.0, 6.0 and 7.0 mg/mL |
| | Ultra turrax-ultrasound | With and without |
| NE III | Na-CMC concentration | 0.2, 0.4, 0.8, 1.2, 1.6, 2.0 mg/mL |

**Table S2.** Particle size (PS) of NE II for increasing concentration of low and medium molecular weight chitosan (LMW and MMW, respectively).

| Chitosan concentration (mg/mL) | PS (nm)* | | |
|---|---|---|---|
| | LMW Chitosan | MMW Chitosan | $p$-value |
| 0.12 | 205.86 ± 0.48 | 226.90 ± 50.57 | $4.05 \times 10^{-1}$ |
| 0.16 | 205.68 ± 11.28 | 229.02 ± 60.73 | $5.90 \times 10^{-1}$ |
| 0.20 | 201.01 ± 37.64 | 230.00 ± 5.58 | $1.41 \times 10^{-3}$ |
| 0.40 | 251.35 ± 47.77 | 232.24 ± 52.05 | $3.35 \times 10^{-2}$ |
| 0.80 | 262.74 ± 33.58 | 218.28 ± 44.68 | $2.03 \times 10^{-1}$ |
| 1.20 | 206.75 ± 69.62 | 347.82 ± 91.65 | $3.52 \times 10^{-1}$ |
| 1.60 | 373.80 ± 24.21 | 189.56 ± 26.88 | $8.79 \times 10^{-4}$ |
| 2.00 | 227.98 ± 11.39 | 392.30 ± 125.33 | $1.52 \times 10^{-1}$ |
| 3.00 | 292.70 ± 59.58 | 226.42 ± 64.93 | $5.19 \times 10^{-3}$ |
| 4.00 | 218.43 ± 180.99 | 399.12 ± 134.76 | $6.82 \times 10^{-1}$ |
| 5.00 | 379.90 ± 57.57 | 195.36 ± 107.33 | $6.50 \times 10^{-3}$ |
| 6.00 | 335.20 ± 57.25 | 224.16 ± 162.17 | $7.26 \times 10^{-2}$ |
| 7.00 | 335.20 ± 34.76 | 187.42 ± 73.15 | $3.79 \times 10^{-3}$ |
| $p$-value | $3.51 \times 10^{-11}$ | $1.01 \times 10^{-9}$ | |

* The values are presented as the average of five true replicates ± standard deviation.

**Table S3.** Effect of Na-CMC concentration on the particle size (PS), polydispersity index (PDI) and zeta potential (ZP) of NE III.

| Na-CMC Concentration (mg/mL) | PS (nm)* | PDI* | ZP (mV)* |
|---|---|---|---|
| 0.2 | 246.68 ± 81.20 | 0.495 ± 0.068 | 21.64 ± 2.72 |
| 0.4 | 221.68 ± 13.50 | 0.294 ± 0.063 | 18.12 ± 2.84 |
| 0.8 | 268.80 ± 63.17 | 0.405 ± 0.052 | 13.10 ± 1.65 |
| 1.2 | 258.52 ± 40.45 | 0.640 ± 0.037 | 9.90 ± 0.38 |
| 1.6 | 345.02 ± 72.43 | 0.627 ± 0.097 | 2.50 ± 2.08 |
| 2.0 | 306.08 ± 92.66 | 0.769 ± 0.033 | −6.40 ± 2.42 |
| *p*-value | $4.89 \times 10^{-2}$ | $7.86 \times 10^{-10}$ | $2.36 \times 10^{-13}$ |

* The values are presented as the average of five true replicates ± standard deviation.

**Table S4.** Particle size (PS), polydispersity index (PDI) and zeta potential (ZP) of loaded NE III during time at 4 and 20 °C.

| Time (days) | PS (nm)* | | PDI* | | ZP (mV)* | |
|---|---|---|---|---|---|---|
| | **20 °C** | **4 °C** | **20 °C** | **4 °C** | **20 °C** | **4 °C** |
| 1 | 324.21 ± 0.97 | 324.23 ± 0.86 | 0.64 ± 0.09 | 0.64 ± 0.14 | −0.95 ± 2.68 | −0.96 ± 3.14 |
| 3 | 529.78 ± 5.52 | 513.94 ± 3.56 | 0.73 ± 0.14 | 0.71 ± 0.10 | 3.88 ± 3.01 | 3.61 ± 2.21 |
| 5 | 735.64 ± 26.88 | 703.77 ± 10.24 | 0.87 ± 0.16 | 0.80 ± 0.13 | 8.21 ± 5.22 | 8.14 ± 2.73 |
| 7 | 870.16 ± 44.67 | 754.22 ± 22.30 | >1 | >1 | 10.74 ± 1.07 | 12.27 ± 4.31 |
| 14 | >1000 | >1000 | >1 | >1 | 1.23 ± 2.51 | 5.09 ± 4.76 |
| 21 | >1000 | >1000 | >1 | >1 | −11.61 ± 1.33 | −15.31 ± 1.15 |

* The values are presented as the average of five samples ± standard deviation. .
